# Spinal Anesthesia in Hemodynamic Instability: A Case Report

**DOI:** 10.7759/cureus.33821

**Published:** 2023-01-16

**Authors:** Luciana Lopes, Joana Marialva, Helder Cardoso

**Affiliations:** 1 Department of Anesthesia, Centro Hospitalar do Tâmega e Sousa, Penafiel, PRT

**Keywords:** contraindication, medical dilemma, continuous spinal anesthesia, prophylactic norepinephrine, hemodynamic instability

## Abstract

Anesthetic dilemmas are not rare in daily practice. Frequently, patients present with comorbid conditions that make general anesthesia risky (e.g., difficult airway and severe pulmonary dysfunction) and contraindications to neuraxial anesthesia at the same time. Reports on the successful anesthetic management of these patients can provide useful information.

We report a case of a patient with severe hemodynamic instability who underwent spinal anesthesia for surgical hip debridement. General anesthesia and airway manipulation were avoided because the patient had recently recovered from SARS-CoV-2 pneumonia amid the first wave of the coronavirus disease 2019 (COVID-19) pandemic when very little was known about the disease and no ventilators were available for postoperative care.

We explain in detail the continuous spinal anesthesia technique using a conventional epidural catheter and prophylactic norepinephrine when cardiovascular instability was the major concern.

## Introduction

Neuraxial anesthesia has many advantages, such as avoiding airway manipulation and reducing the incidence of pulmonary complications [1]. Depending on the surgical site, it is an alternative to general anesthesia in patients with difficult airways and severe pulmonary disease [2]. However, hemodynamic instability is a commonly cited contraindication to neuraxial anesthesia.

In the early phases of the coronavirus disease 2019 (COVID-19) pandemic, the consequences of general anesthesia and positive ventilatory support after recent COVID-19 pneumonia were unknown, and regional anesthesia was recommended whenever possible.

Our report describes the successful management of neuraxial anesthesia in a patient with hemodynamic instability, in whom general anesthesia was considered risky. This report does not provide evidence that regional anesthesia was the better option. It describes the successful management of neuraxial anesthesia in a setting of severe cardiovascular instability.

## Case presentation

An 83-year-old female with an American Society of Anesthesiologists (ASA) score of III and a history of left-sided hemiparesis, hypertension, and diabetes was admitted to our hospital for surgical repair of a recent and recurrent dislocation of her hip replacement prosthesis. However, the surgery had to be postponed because of an unknown SARS-CoV-2 infection diagnosed at the time of admission.

After she recovered from mild SARS-CoV-2 pneumonia, for which she received low oxygen flow at a rate of 2 L/minute by nasal cannula for 15 days, while waiting in the ward for the scheduled orthopedic surgery, signs of a new infection manifested, and an ultrasound revealed a periprosthetic fluid collection. The situation evolved in 24 hours into a hemodynamic shock, and urgent surgical hip debridement and irrigation were decided.

During the preoperative evaluation, the patient was hypotensive (64/30 mmHg), had a normal heart rate (85 bpm), was tachypneic (respiratory rate: 25 bpm), and was pyretic (temperature: 38°C/100.4°F). The patient had a depressed consciousness (Glasgow Coma Scale score: 9), poor peripheral perfusion, and skin mottling in the extremities. Cardiac and pulmonary auscultation findings were normal. The last chest radiograph showed normal findings, but a new chest X-ray was not ordered to avoid delaying resuscitation. The laboratory results are shown in Table 1.

**Table 1:** Laboratory results PCO_2_: arterial carbon dioxide pressure; HCO_3-_: plasma bicarbonate levels; PaO_2_: arterial oxygen pressure; Na^+^: sodium plasma levels; K+: potassium plasm levels; Cl^-^: chloride plasma levels

Parameter	Patient results	Normal range
Arterial pH	7.32	7.35-7.45
PCO_2_	33 mmHg	35-45 mmHg
HCO_3-_	20 mmol/L	22-26 mmol/L
PaO_2_	65 mmHg	75-100 mmHg
Lactate levels	2.7 mmol/L	0.5-2 mmol/L
Creatinine	1.4 mg/dL	0.7-1.3 mg/dL
Urea	184 mg/dL	6-24 mg/dL
C-reactive protein	60 mg/dL	<5 mg/L
White blood cell count	12.7 × 10^9^/L	4.5-11 × 10^9^/L
Na^+^	154 mEq/L	135-145 mEq/L
K+	5.1 mEq/L	3.6-5.2 mEq/L
Cl^-^	98 mEq/L	96-106 mEq/L
Ionized Ca^2+^	1.08 mmol/L	1.2-1.32 mmol/L
Albumin	5.1 g/L	3.4-5.4 g/L
Albumin corrected anion gap	44.7	8-12
Hemoglobin	10.2 g/dL	11.6-15 g/dL
Platelet count	154 × 10^9^/L	150-450 × 10^9^/L
Activated partial thromboplastin time	32 seconds	25-35 seconds
Prothrombin time	11.2 seconds	11-13.5 seconds

Clinical findings, laboratory tests, and imaging results suggested that the cause of hemodynamic instability was probably a combination of hypovolemia and sepsis.

Due to the COVID-19 pandemic hustle and exhausting intensive care resources, the unstable patient was urgently taken from the general ward to the operating room where she was resuscitated, anesthetized, and surgically treated. Logistical and clinical concerns of unpredictable pulmonary complications caused by general anesthesia, intubation, and positive ventilation led the multidisciplinary team composed of anesthesiologists and intensive care physicians to consider regional anesthesia as the best option.

Hemodynamic resuscitation was rapidly achieved using intravenous fluids and norepinephrine. Due to concerns about hemodynamic collapse after neuraxial anesthesia, norepinephrine infusion was prophylactically increased just before a continuous spinal block was titrated.

The most important steps were as follows (some performed simultaneously). (1) Intravenous norepinephrine was titrated to a mean blood pressure of ≥65 mmHg, and approximately 30 mL/kg of crystalloid solution was administered. Before central venous access, norepinephrine was infused at a concentration of 100 µg/mL by the peripheral vein to avoid delayed resuscitation. (2) Blood was drawn for cultures and laboratory studies, antibiotics were administered, arterial and central venous lines were inserted, and a bladder catheter was placed. (3) A conventional epidural catheter was inserted into the subarachnoid space (Portex® Epidural Kit; Smiths Medical, Minneapolis, MN, USA) at the level of L3-L4 with a Tuohy 18-G needle via a paramedian approach. After a free flow of cerebrospinal fluid, about 2 cm of the epidural catheter was introduced into the subarachnoid space. (4) Cerebrospinal fluid was aspirated through the catheter to confirm the correct intrathecal positioning and remove the air present in the catheter and filter. (5) An anesthetic mixture was prepared for subarachnoid administration, constituted by 2 mL levobupivacaine 0.5% + 0.5 mL sufentanil (final composition per milliliter: 3.33 mg levobupivacaine and 0.8 µg of sufentanil). (6) The “dead space” of the catheter and filter was filled with the anesthetic mixture (approximately 0.9 mL).

Immediately before the spinal block, the norepinephrine infusion rate was prophylactically increased to raise the mean blood pressure by approximately 30% (≥85 mmHg). Subsequently, 1 mL of the anesthetic mixture was administered into the subarachnoid space through the catheter, and the administration of 1 mL was repeated five minutes later (total dose: 6.66 mg levobupivacaine + 1.6 µg sufentanil). The handover of the patient to the surgical team took approximately 50 minutes, and the surgery took approximately one hour. There was no need to repeat the anesthetic dose.

After resuscitation and spinal anesthesia, no hypotension was observed. Hemodynamic stability was observed throughout the study. Urinary output and improved consciousness were observed before the end of surgery. In the postoperative period, the patient was admitted to a high-dependency unit with unassisted spontaneous breathing, conscious state, and sluggish response to simple orders. She required low-dose norepinephrine over the following 48 hours and was transferred to the ward on the third postoperative day.

## Discussion

It is noteworthy that the purpose of this report is not to discuss the appropriateness of one anesthetic technique over the other but to report how we managed severe cardiovascular instability once the decision of administering regional anesthesia was made. From this perspective, useful information can be obtained from the case. It is important to emphasize again that contraindications for neuraxial anesthesia vary widely in their categorization, between absolute and relative, depending on the source. However, despite the reasons that led to the avoidance of general anesthesia, the authors recognize that the option for regional anesthesia may be a subject of reasonable dispute. The decision to perform a regional anesthetic technique was made on an individual basis, considering the global risks and benefits, and the local logistic conditions.

The most frequent causes of shock during anesthesia are hypovolemia, sepsis, cardiac failure, and circulatory obstruction, and patients may present with a combination of these. Hemodynamic instability in our patient was probably due to a combination of sepsis and true hypovolemia. Our elderly patient recovered in an overcrowded hospital when vigilance was lower than desirable and the inability to ingest water adequately was a risk. Unrecognized hypovolemia and hip infection caused the cardiovascular failure. Current guidelines for sepsis management recommend immediate resuscitation and surgical intervention within the first 6-12 hours [3]. However, amid a pandemic peak wave in a resource-exhausted hospital, it was considered best to resuscitate and initiate surgery as soon as possible when the patient arrived in the operating room.

General anesthesia and orotracheal intubation would have been the best anesthetic techniques for our patient; however, general anesthesia may be challenging and unpredictable in patients with severe pulmonary disease [2].

Neuraxial anesthesia has well-known contraindications that vary in categorization between absolute and relative depending on the bibliographic source. Absolute contraindications may include patient refusal, local infection at the puncture site, and allergy to any of the drugs to be administered, and relative contraindications may comprise neuropathy (central or peripheral), spinal stenosis, previous spinal surgery, multiple sclerosis, spina bifida, coagulopathy (inherited or acquired), aortic stenosis, fixed cardiac output, hypovolemia, and systemic infection (including septic shock) [4].

In the early months of the pandemic, very little was known about the consequences of SARS-CoV-2 pneumonia, which is why general anesthesia and intubation were avoided. The fact that no ventilators were available for postoperative treatment contributed to this decision. The anesthesia team chose a regional technique being aware of its significant controversial aspects, such as spinal puncture in a patient with a depressed level of consciousness being unable to report pain or paresthesia during the block, inability to confirm adequate preoperative sensory anesthesia, risk of pulmonary aspiration, risk of neuraxial hematoma, risk of neuraxial infection, and significant risk of hemodynamic collapse in the context of cardiovascular dysfunction caused by sepsis and hypovolemia.

Respiratory concerns were reduced to some extent because the patient had acceptable preoperative spontaneous ventilatory dynamics and satisfactory blood gas values, and the entire procedure could be performed in the lateral decubitus position. If our patient required general anesthesia during surgery, it would have been administered without delay, and if there was a need for continuing postoperative ventilatory support, the operating room would have been converted to fulfill this demand.

Regarding coagulopathy and bacteremia due to sepsis, normal coagulation tests and administration of antibiotics before the neuraxial approach lessened the concerns to some extent. Available data suggest that patients with evidence of systemic infection may safely undergo spinal anesthesia, provided appropriate antibiotic therapy is initiated before dural puncture and the patient has shown a response to therapy. However, “placement of an indwelling epidural (or intrathecal) catheter in this group of patients remains controversial” [5].

Regarding cardiovascular instability, the sequence of the procedural steps was based on the anticipation of predicted pathophysiological derangements, specifically vasodilation, myocardial depression, hypovolemia (attributable to sepsis and hypovolemia), and sympathectomy (attributable to spinal block). Hemodynamic changes that occur with spinal anesthesia are caused by sympathetic blockade, which induces venous and arterial vasodilation, and possible blockade of cardio-accelerator fibers from T1 to T4 [6]. To antagonize these changes, norepinephrine may be the best amine because of its alfa-1 and beta-autonomic activity. It is also the first-line vasoconstrictor for the treatment of septic shock and has been previously used for prophylaxis of hypotension after spinal anesthesia [4]. A more objective means of determining the patient’s volume status, such as the arterial line waveform analysis, esophageal Doppler, or echocardiography, would have been useful; however, this was not possible or available.

Despite its increasing use, peripherally administered norepinephrine may be a subject of discussion. The concentration used in this patient (100 µg/L) should have been lower. Publications on this subject make some recommendations, such as the use of concentrations between 16 and 32 µg/L, durations not exceeding 6-12 hours, intravenous access gauge size between 18 and 20 G, locations proximal to (or at) the antecubital fossa, monitoring site every two hours, a de-escalation plan to wean, and phentolamine or nitroglycerin paste available in case extravasation occurs [7]. We justified our decision of infusing highly concentrated peripheral norepinephrine considering the urgency of the situation until central venous access was in place.

**Figure 1:**
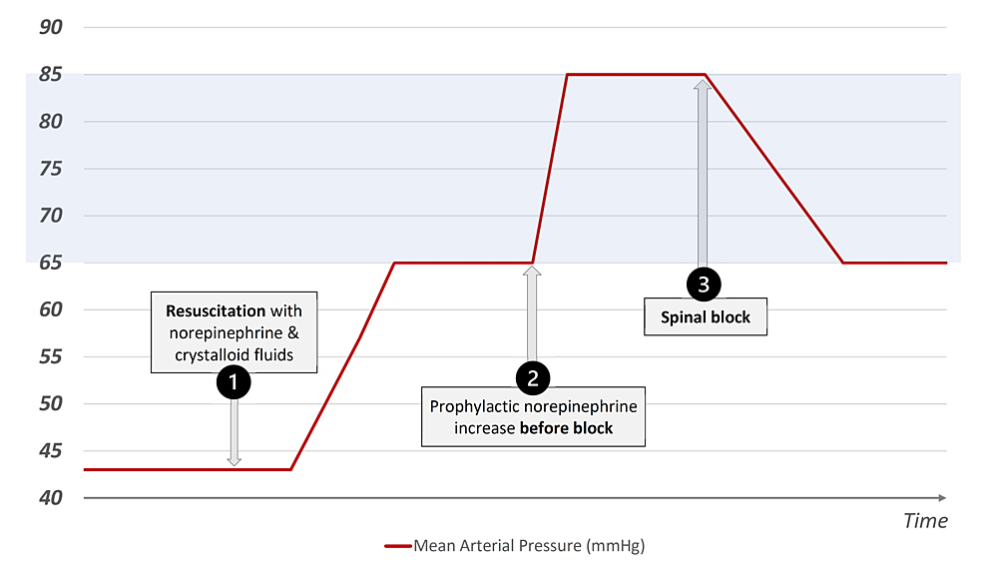
Therapeutic interventions and mean arterial pressure (1) First, resuscitation was accomplished with norepinephrine and crystalloid fluids to a mean arterial pressure of 65 mmHg. After patient stabilization, the norepinephrine infusion was prophylactically increased to a mean arterial pressure of 85 mmHg (2) to accommodate the ensuing hypotension provoked by the spinal block (3). Figure courtesy by the author H. Cardoso

In our case, it was important to first correct the hypovolemia and the sepsis-related hemodynamic consequences with norepinephrine and fluids until a mean arterial blood pressure of 65 mmHg was achieved. Before the continuous spinal block was initiated, norepinephrine infusion was prophylactically augmented to increase the mean arterial blood pressure from ≥65 mmHg to ≥85 mmHg (approximately 30%). The rationale for the prophylactic increase in mean blood pressure of 30% was to obtain a safety margin in case of hypotension caused by spinal sympathectomy [8]. This is because mean blood pressure decreases between 15% and 33% if the cardiac output does not decrease after spinal anesthesia (Figure 1) [6].

Discussing the option for continuous spinal anesthesia, it is not a technique frequently executed because of the fear of post-dural puncture headache and the risk of cerebrospinal fluid fistula, and it is technically more demanding and time-consuming than single-shot anesthesia. However, continuous spinal anesthesia has many advantages, including the ability to confirm the correct intrathecal catheter tip position (through aspiration of cerebrospinal fluid), rapid onset (very important for the anesthesiologist in urgent situations), denser block, and the possibility of incremental doses as needed. Moreover, continuous spinal anesthesia may confer more excellent hemodynamic stability than single-shot anesthesia [9]. The fact that continuous spinal anesthesia can be performed using conventional epidural catheters is very convenient because the technique is more familiar to the anesthetist and commercial kits are ubiquitous in any operating room, even in low-resource settings.

Epidural anesthesia was also considered. Some of the advantages of epidural catheter placement against continuous spinal anesthesia are the avoidance of disruption of the dural barrier, which may be protective against the spread of bacteria into the intrathecal space, decreased risk of cerebrospinal fluid fistula, and the decreased risk of post-dural puncture headache. However, the disadvantages are the impossibility of confirming the right tip placement, less dense block, very slow unset in urgent situations, and the more unpredictable volume of local anesthetic solution needed to robustly (but solely) cover the lower spinal segments.

## Conclusions

Often, regional anesthesia is a desirable option; however, cardiovascular instability frequently hinders its choice. This report may suggest a strategy for the management of neuraxial anesthesia when the cardiovascular risk is high and general anesthesia is undesirable or impractical (e.g., severe pulmonary dysfunction). However, understandably, the cumulative contraindications, although relative, present in our case make it susceptible to reasonable objections.

In summary, a conventional epidural catheter in the subarachnoid space and a prophylactic norepinephrine infusion allowed neuraxial anesthesia in a patient with severe cardiovascular instability.

Further controlled clinical studies are needed to assess the risks and benefits of continuous neuraxial anesthesia combined with prophylactic noradrenaline in selected patients with a very high cardiovascular risk.
